# Etomidate Monotherapy for Perioperative Control of Hypercortisolism in a Mitotane-Intolerant Patient With Adrenocortical Carcinoma: A Case Report

**DOI:** 10.7759/cureus.115808

**Published:** 2026-09-05

**Authors:** Naeem Alkheamy, Jalal Jbara, Nissar Shaikh, Umm E Amara, Umme Nashrah, Shahzad Anjum, Gamal Al-Ameri

**Affiliations:** 1 Medical Education, Hamad Medical Corporation, Doha, QAT; 2 Faculty of Medicine and Health Sciences, UCSI (University College Sedaya International) University, Kuala Lumpur, MYS; 3 Anesthesiology, Intensive Care Unit and Perioperative Medicine, Hamad Medical Corporation, Doha, QAT; 4 Surgical Intensive Care, Hamad Medical Corporation, Doha, QAT; 5 Obstetrics and Gynecology, Deccan College of Medical Sciences, Hyderabad, IND; 6 Medicine, Deccan College of Medical Sciences, Hyderabad, IND; 7 Emergency Medicine, Hamad Medical Corporation, Doha, QAT

**Keywords:** acth, adrenocortical carcinoma (acc), etomidate, mitotane, cushing’s syndrome

## Abstract

Endogenous Cushing syndrome is a metabolic syndrome that results from the excessive production of cortisol from the adrenal gland. Hypercortisolism can result in serious complications, such as hypertension, myocardial ischemia, glucose intolerance, immunosuppression, and electrolyte disturbances. The mainstay of treatment is surgical resection for resectable tumors, along with pharmacological therapy in selected cases, because severe hypercortisolism often requires prompt medical stabilization before definitive surgical management can be safely performed.

We report a 34-year-old male patient who presented with generalized weakness, peripheral numbness, and palpitations and was found to have severe hypokalemia, metabolic alkalosis, and high levels of cortisol. Diagnostic laboratory tests showed adrenocorticotropic hormone (ACTH)-independent Cushing syndrome, which was confirmed with magnetic resonance imaging (MRI) and positron emission tomography (PET) imaging. Etomidate was used to control hypercortisolism preoperatively as a bridge to definitive surgical resection of right adrenal adenocarcinoma after failure of a course of mitotane therapy. Sub-anesthetic doses of etomidate dramatically lowered cortisol levels, reduced the need for potassium replacement, and controlled blood pressure with fewer side effects.

## Introduction

Cushing syndrome is a rare endocrine disorder resulting from cortisol overproduction. The annual incidence is two to three cases per million, of which the female-to-male ratio is 3:1 [1]. Cortisol is a steroid hormone that is produced by the adrenal zona fasciculata from its precursor, cholesterol, through a multistep reaction. Cortisol exerts its effects by binding to its intracellular glucocorticoid receptor, which is present in all body organs. The cortisol-receptor complex then enters the nucleus of the cell and affects gene transcription. The production of cortisol is well and tightly controlled by the hypothalamic-pituitary-adrenal axis [2,3]. Disruption of this axis by a functioning tumor might result in cortisol overproduction, resulting in a typical Cushingoid appearance, including central obesity, moon face, and the presence of purple striae, with multisystem manifestations such as impaired glucose metabolism, high blood pressure, muscle wasting, osteoporosis, psychosis, and depression.

Etomidate is an imidazole derivative that is currently used primarily for the induction of general anesthesia. The initial report on etomidate was published in 1965, when it was first developed as an antifungal medication. It was noted that etomidate metabolites cause significant sedation by targeting GABA receptors in the central nervous system, and it was subsequently introduced for this purpose in the early 1970s [4]. With growing use, several side effects have been reported, including adrenal gland suppression, which was first reported in 1983 [5]. Etomidate can cause adrenal suppression by inhibiting 11β-hydroxylase, an important steroidogenic enzyme involved in cortisol synthesis. Etomidate has been described in several case reports and retrospective studies as being useful in the treatment of hypercortisolism.

This case report provides evidence to support the use of etomidate as a potential alternative to mitotane when mitotane causes liver derangements or is contraindicated. We report a case in which etomidate was used as monotherapy to control hypercortisolism in the perioperative period after failure of treatment with another line of therapy.

## Case presentation

A 34-year-old man with morbid obesity (BMI 42 kg/m²) and a history of childhood asthma presented to the Emergency Department with generalized weakness, bilateral hand numbness, and intermittent palpitations. He had also gained approximately 12 kg over the previous six months. His blood pressure on presentation was 180/112 mmHg, while the remaining vital signs were within normal limits. Examination revealed moon facies and abdominal striae. The initial laboratory investigations are summarized in Table 1.

**Table 1:** Initial laboratory investigations

Parameter	Patient Value	Reference Range
pH	7.61	7.35-7.45
Serum bicarbonate	48 mmol/L	22-29 mmol/L
Potassium	2.0 mmol/L	3.5-5.3 mmol/L
Magnesium	0.86 mmol/L	0.7-1 mmol/L
Creatinie	35 umol/L	62-106 umol/L
Soduim	140 mmol/L	133-146 mmol/L
High-sensitivity troponin	71 ng/L	3-15 ng/L
Morning serum cortisol	1115 nmol/L	138-689 nmol/L
Adrenocorticotropic hormone (ACTH)	<1.5 pg/mL	7.2-63.3 pg/mL
Glucose	13.5 mmol/L	3.3-5.5 mmol/L

High-sensitivity troponin was 71. ECG demonstrated left axis deviation with flattened T waves, whereas echocardiography was normal. Coagulation profile, thyroid function tests, and chest X-ray were also unremarkable. Based on the clinical findings, Cushing syndrome was suspected. Because of severe hypokalemia with ECG changes, the patient was admitted to the Intensive Care Unit for potassium replacement. He was transferred to the medical ward two days later. Further investigations confirmed adrenocorticotropic hormone (ACTH)-independent Cushing syndrome, with a morning cortisol level of 1115 nmol/L (reference range, 138-689 nmol/L), ACTH <1.5 pg/mL, and a positive dexamethasone suppression test. Twenty-four-hour urinary catecholamines and metabolites were 4.5 μmol/24 h, excluding pheochromocytoma. Magnetic resonance imaging (MRI) demonstrated multiple fat-containing solid lesions arising from the medial and lateral aspects of the right adrenal gland, measuring up to 10 cm, together with multiple fat-containing aortocaval and retrocaval lesions at the renal level, measuring up to 28 mm (Figure 1).

**Figure 1:**
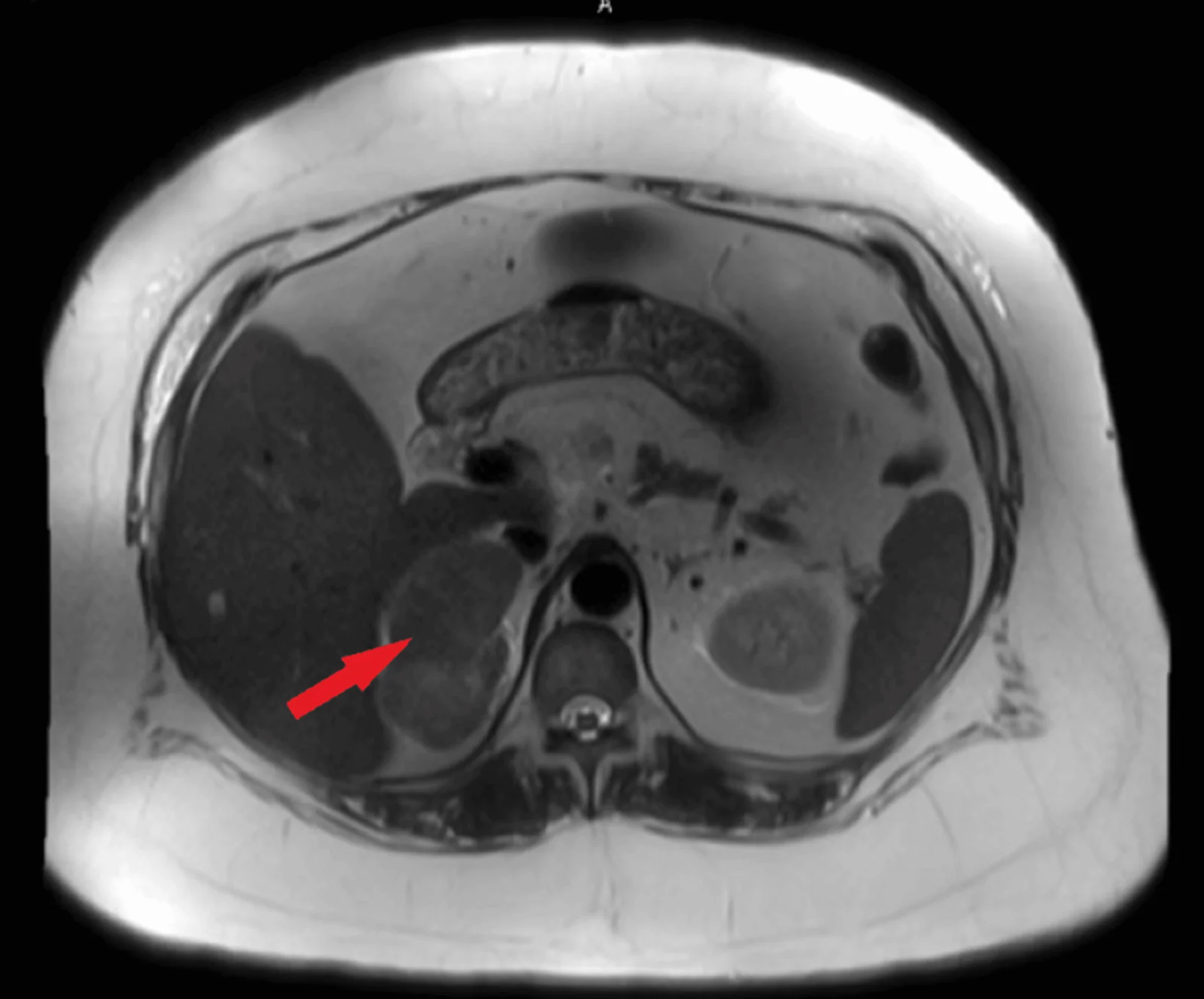
Abdominal MRI showing right adrenal solid lesion (arrow) MRI: magnetic resonance imaging

Positron emission tomography (PET) scan showed intense fluorodeoxyglucose (FDG) uptake in the right adrenal gland mass lesion and para-aortic lymph nodes, suggestive of adrenocortical carcinoma with metastases (Figure 2).

**Figure 2:**
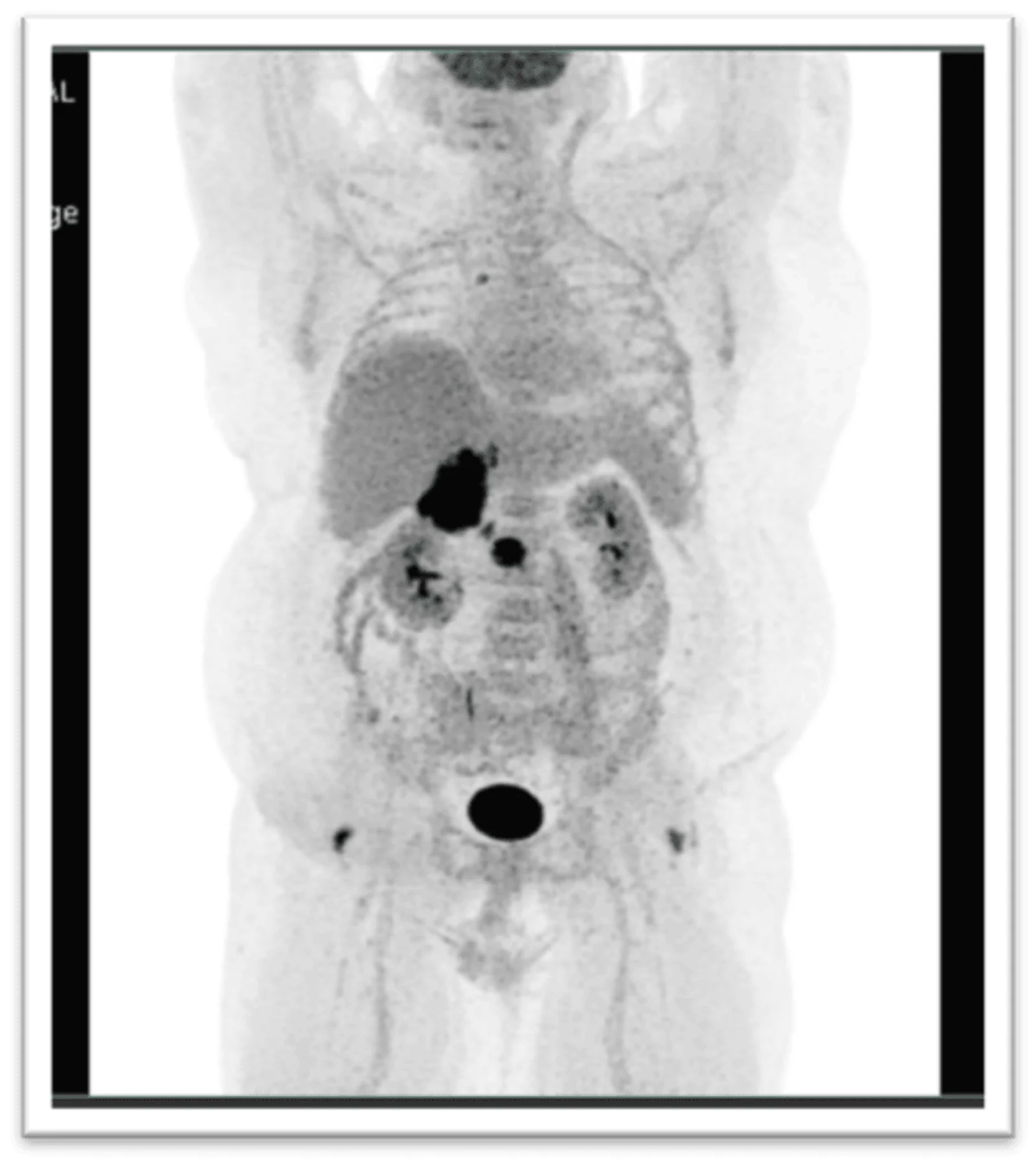
Whole-body FDG PET/CT demonstrating intense FDG uptake in the right adrenal and para-aortic lesions FDG PET/CT: fluorodeoxyglucose positron emission tomography/computed tomography

Computed tomography (CT)-guided biopsy of the adrenal mass was performed, but the result was inconclusive, showing adenoma versus adenocarcinoma. The multidisciplinary team decided to proceed with debulking surgery. The hospital course was significant for resistant hypokalemia requiring frequent potassium replacement, hyperglycemia requiring insulin, and high blood pressure requiring two antihypertensive medications.

The patient was transferred to the Urology Ward for preoperative optimization. Surgery was delayed twice because of electrolyte derangement and an episode of chest infection with asthma exacerbation requiring noninvasive ventilation and admission to the surgical intensive care unit (SICU). Due to the markedly elevated cortisol levels, resistant hypokalemia, and the need to optimize the patient for surgery, mitotane chemotherapy was initiated at a dose of 500 mg three times daily. However, there was no response, and the patient developed deranged liver enzymes (aspartate aminotransferase (AST) 106 U/L, alanine aminotransferase (ALT) 188 U/L, alkaline phosphatase (ALP) 379 U/L, total bilirubin 167 μmol/L); therefore, mitotane was discontinued after four days. While in the SICU, the multidisciplinary team agreed to initiate etomidate for the management of hypercortisolism. Etomidate 5 mg IV was administered slowly over 10 minutes, followed by a continuous infusion at 0.02 mg/kg/hour based on lean body weight, with incremental dose adjustments of 0.01-0.02 mg/kg/hour according to serum cortisol levels. The target cortisol level was 300-500 nmol/L. Serum cortisol was monitored every six hours, and potassium was monitored daily. Following initiation of etomidate, serum cortisol levels progressively decreased from 1790 nmol/L on Day 1 to 1550, 1100, and 500 nmol/L on Days 2-4, respectively. Cortisol levels subsequently remained between 260 and 370 nmol/L through Day 10, with the target range of 300-500 nmol/L achieved within four days of treatment. Serum potassium was 4.09 mmol/L on Day 1 and subsequently fluctuated between 2.72 and 5.00 mmol/L during the treatment period, with values of 4.14 mmol/L on Day 8 and 4.01 mmol/L on Day 10; it then stabilized after Day 16. The patient remained in the SICU because of the potential hypnotic effects of etomidate. During the preoperative period, hypertension was controlled with medication, including perindopril 5 mg daily and spironolactone 100 mg daily, while etomidate was titrated according to cortisol levels. Pain was managed with morphine and paracetamol.

Trends in serum cortisol and potassium are shown in Figures 3-4.

**Figure 3:**
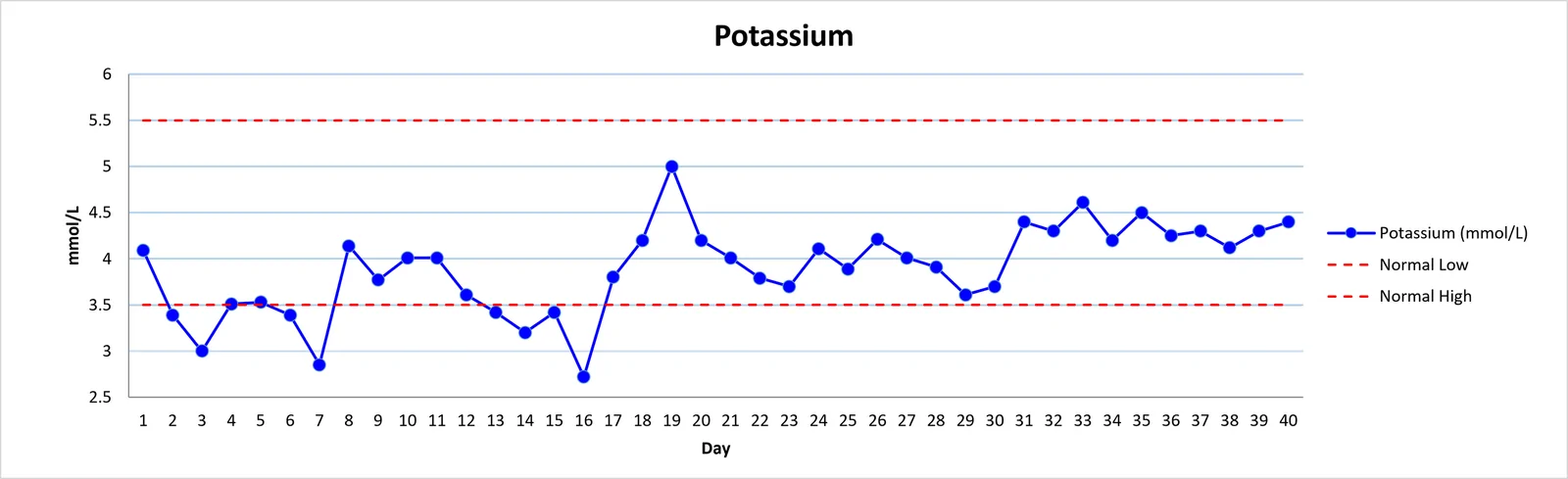
Potassium trend before and after initiation of etomidate on Day 7

**Figure 4:**
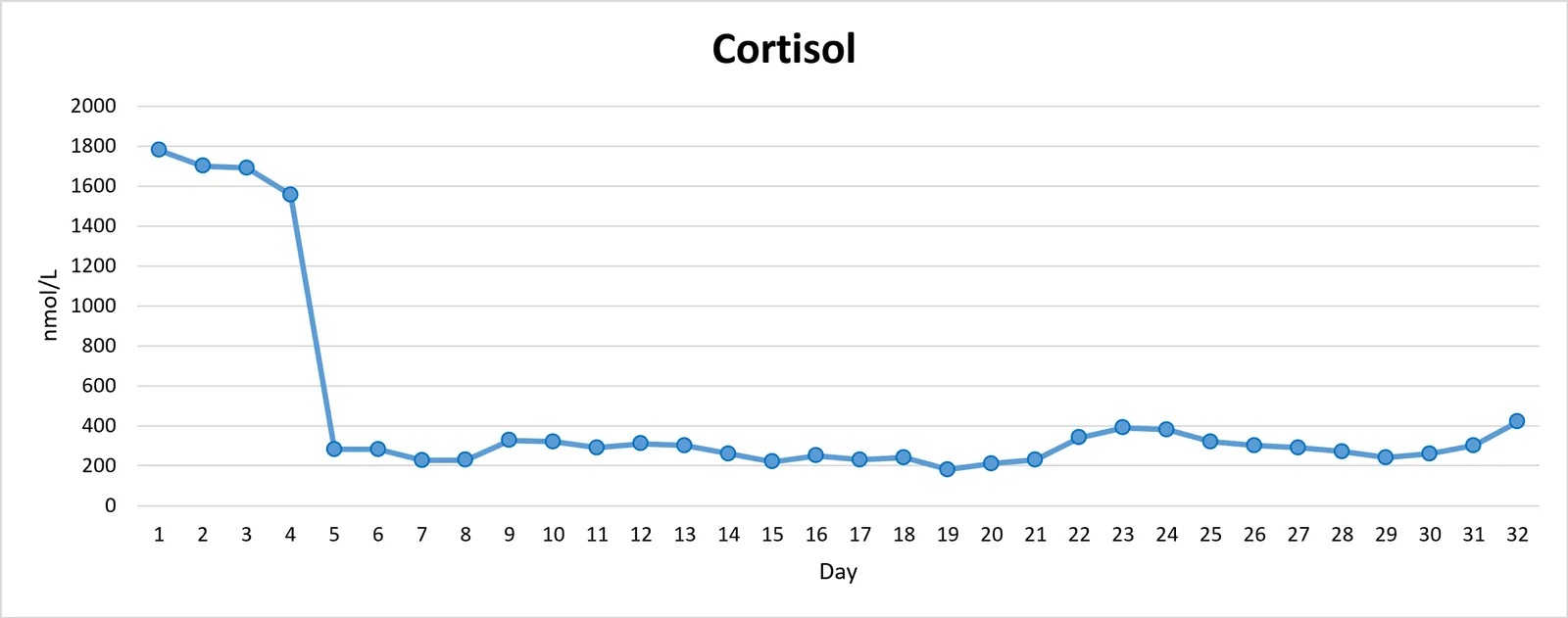
Cortisol trend before and after initiation of etomidate on Day 4

The patient subsequently underwent surgery under general anesthesia. The operation lasted three hours and was uneventful, with easy intubation and only a small dose of norepinephrine infusion required intraoperatively. Anesthetic medications included midazolam 2 mg, fentanyl 507 μg, etomidate 30 mg for induction, and rocuronium 118 mg. Intraoperative fluid administration included 2500 mL of crystalloids and 500 mL of colloids, with a urine output of 350 mL and estimated blood loss of 100 mL. Hydrocortisone 100 mg IV was administered at the end of surgery. The patient was kept intubated, mechanically ventilated, and sedated, and was transferred to the SICU. The last serum cortisol level was 420 nmol/L, at which point the etomidate infusion was discontinued after surgery. Sodium was 130 mmol/L, and potassium was 4.2 mmol/L. A few hours later, the patient was extubated while fully awake, with full motor recovery. Following extubation, he developed bronchospasm and desaturation, which resolved promptly with continuous positive airway pressure (CPAP) and bronchodilators.

## Discussion

Cushing syndrome can increase mortality fourfold, which is mainly attributed to cardiovascular events [6]. Most cases of ACTH-independent Cushing syndrome are attributed to unilateral adrenal adenoma, followed by adrenocortical carcinoma [7]. Patients with Cushing syndrome due to adrenal carcinoma are at increased risk of complications and have a poor prognosis [8,9]. Complications of Cushing syndrome include an increased risk of infections, cardiovascular events, impaired glucose metabolism, osteoporosis, psychosis, and electrolyte imbalance. The presentation of Cushing syndrome might vary from overt Cushing syndrome to subclinical hypercortisolism. Diagnosis is challenging and requires multiple steps, as well as timely and extensive laboratory and radiological investigations [10]. Immediate treatment may be warranted and includes identifying and correcting electrolyte and acid-base disturbances, controlling blood pressure and glucose levels, and treating infection if present. The first-line therapy is surgical resection of the hyperfunctioning lesion. Pharmacological treatment is usually required for patients who need rapid control of hypercortisolism, including patients with acute psychosis, those in the perioperative period, or those who are not candidates for resection or have persistent high cortisol levels despite surgery due to recurrence [11].

Steroidogenesis inhibitors such as mitotane and ketoconazole are the mainstays of medical treatment and can be used as monotherapy or in combination, with variable success rates, significant drug-drug interactions, and adverse events reported across several studies [12-14]. Mitotane is a first-line therapy to control hypercortisolism secondary to adrenocortical carcinoma. In this case, mitotane 500 mg three times daily did not reduce the cortisol level and was discontinued due to liver toxicity after four days of therapy. Etomidate has been described in several case reports and studies as a useful and effective cortisol-lowering agent, regardless of lesion location or the timing of initiation in relation to surgery. The recommended regimen is a loading dose of 3-5 mg, followed by a continuous infusion of 0.03-0.10 mg/kg/h (2.5-3.0 mg/h) [9].

Controversy does exist regarding the dose and duration of etomidate and the desired cortisol level. Several studies have shown that even lower doses of etomidate can achieve the desired cortisol level [15,16]. On the other hand, block-and-replace is an alternative strategy, but it requires a higher etomidate dose and steroid replacement therapy [16]. In this case report, etomidate was started and titrated to a target cortisol level of 200-500 nmol/L, which was quickly achieved within a few hours without hypnotic side effects. Advantages and indications of each strategy are still lacking. Moreover, a few reports have described prolonged use of etomidate for 2.5 to 5.5 months in two critically ill patients [15,16]. In this case, we used etomidate for six days as a bridge to surgery and stopped it immediately postoperatively. A disadvantage of etomidate use is the need for an IV infusion to control cortisol levels, which can significantly reduce patient mobilization.

## Conclusions

Etomidate may be a useful option for the rapid control of severe hypercortisolism in the perioperative period, particularly when other medical therapies are not tolerated or fail to achieve adequate cortisol control. In this case, etomidate was used successfully as a bridge to definitive surgery and allowed cortisol levels to be controlled during the preoperative period. However, its use requires continuous intravenous infusion and close monitoring because of the potential for sedation and other adverse effects. Further studies are needed to better define the optimal dose, duration, and target cortisol level for etomidate therapy.
